# No evidence for female kin association, indications for extragroup paternity, and sex‐biased dispersal patterns in wild western gorillas

**DOI:** 10.1002/ece3.7596

**Published:** 2021-05-25

**Authors:** Shelly Masi, Frédéric Austerlitz, Chloé Chabaud, Sophie Lafosse, Nina Marchi, Myriam Georges, Françoise Dessarps‐Freichey, Silvia Miglietta, Andrea Sotto‐Mayor, Aurore San Galli, Ellen Meulman, Emmanuelle Pouydebat, Sabrina Krief, Angelique Todd, Terence Fuh, Thomas Breuer, Laure Ségurel

**Affiliations:** ^1^ UMR7206 Eco‐anthropologie Muséum national d’Histoire naturelle CNRS Université de Paris; Musée de l'Homme Paris France; ^2^ Department of Biology Ecole normale supérieure PSL University Paris Paris France; ^3^ Department Adaptations du Vivant UMR7179 MECADEV CNRS/MNHN Paris France; ^4^ Dzanga‐Sangha Protected Areas World Wide Fund for Nature Bangui Central African Republic; ^5^ Wildlife Conservation Society Global Conservation Program Bronx NY USA; ^6^ Present address: CMPG Institute for Ecology and Evolution University of Berne Berne Switzerland; ^7^ Present address: UMS2700 2AD ‐ Acquisition et Analyse de Données pour l'Histoire naturelle Concarneau France; ^8^ Present address: Fauna & Flora International Cambridge UK; ^9^ Present address: World Wide Fund for Nature –Germany Berlin Germany; ^10^ Present address: Laboratoire de Biométrie et Biologie Evolutive CNRS ‐ Université de Lyon Villeurbanne France

**Keywords:** dispersal, great apes, kin association, paternity, polygynous species, western gorillas

## Abstract

Characterizing animal dispersal patterns and the rational behind individuals’ transfer choices is a long‐standing question of interest in evolutionary biology. In wild western gorillas (*Gorilla gorilla*), a one‐male polygynous species, previous genetic findings suggested that, when dispersing, females might favor groups with female kin to promote cooperation, resulting in higher‐than‐expected within‐group female relatedness. The extent of male dispersal remains unclear with studies showing conflicting results. To investigate male and female dispersal patterns and extragroup paternity, we analyzed long‐term field observations, including female spatial proximity data, together with genetic data (10 autosomal microsatellites) on individuals from a unique set of four habituated western gorilla groups, and four additional extragroup males (49 individuals in total). The majority of offspring (25 of 27) were sired by the group male. For two offspring, evidence for extragroup paternity was found. Contrarily to previous findings, adult females were not significantly more related within groups than across groups. Consistently, adult female relatedness within groups did not correlate with their spatial proximity inferred from behavioral data. Adult females were similarly related to adult males from their group than from other groups. Using *R*_ST_ statistics, we found significant genetic structure and a pattern of isolation by distance, indicating limited dispersal in this species. Comparing relatedness among females and among males revealed that males disperse farer than females, as expected in a polygamous species. Our study on habituated western gorillas shed light on the dispersal dynamics and reproductive behavior of this polygynous species and challenge some of the previous results based on unhabituated groups.

## Graphical abstract

We found evidence for extra‐group paternity for two offspring. Adult females were not significantly more related within groups than between groups and were similarly related to adult males from their group than from other groups. Comparing relatedness among females and among males revealed that males disperse farer than females.

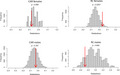

## 1 INTRODUCTION

Sociality, the persistent affiliative association of individuals in groups observed in some animal species, is thought to have evolved for different reasons, one of which is to promote cooperation among kin (e.g., Van Horn et al., 2004). However, even though cooperative behaviors are usually beneficial for individuals (Silk, 2007), the grouping of close relatives can also lead to inbreeding, resulting in high fitness costs (Keller & Waller, 2002; Lukas & Clutton‐Brock, 2011; Pusey & Wolf, 1996). Thus, many species have developed specific behaviors and mating strategies to avoid inbreeding, such as the exclusive or majority dispersal of one sex. In social mammals, male‐biased dispersal is the most commonly observed pattern (Greenwood, 1980; Lawson Handley & Perrin, 2007), as it prevents mating between related individuals while allowing female kin to stay together and cooperate (Kin selection theory: Hamilton, 1964; Silk, 2007). In some mammal species (e.g., equids, bats, primates: Clutton‐Brock & Lukas, 2012), including gorillas (*Gorilla* spp.), natal dispersal (before reproduction) occurs in both sexes. This allows females to avoid mating with their father and brothers (Clutton‐Brock, 1989), and reduces feeding competition by limiting group size (Crockett & Janson, 2000; Vick & Pereira, 1989). In such cases with both male and female dispersal, cooperation between females is rare and female commonly disperse again after natal dispersal (secondary dispersal), as shown, for example, in bats (Debeffe et al., 2015), equids (Nagy et al., 2007), and gorillas (Robbins & Robbins, 2015; Stokes et al., 2003).

While both sexes disperse in the genus *Gorilla*, the two species present different social strategies (Harcourt & Stewart, 2007a; Robbins & Robbins, 2018). Breeding groups of western gorillas (*G. gorilla*) typically include one silverback (Breuer et al., 2010; Gatti et al., 2004; Parnell, 2002), while in eastern gorillas (*G. beringei*), for the well‐studied mountain gorilla, multiple males frequently coexist in a single group (40% of the groups), even though a dominant silverback sires most of the offspring (Bradley et al., 2005). The twice more frequent secondary female dispersal found in western versus eastern gorillas might thus be explained by the greater need of western gorilla females to increase mate choice and reproductive success (Baudouin et al., 2019; Manguette, Robbins et al., 2020; Stokes et al., 2003).

Additionally, western gorillas are seasonal frugivores (Doran et al., 2002; Doran‐Sheehy et al., 2009; Masi et al., 2009) and thus are likely to experience higher feeding competition than the mainly folivorous mountain gorillas. Reliance on monopolizable resources such as fruit may indeed reduce the advantage of cooperative behaviors between females (Wrangham, 1980).

In a mountain gorilla population, a pattern of isolation by distance (i.e., a positive correlation between genetic and geographic distances) was observed for females but not for males, suggesting a larger mean dispersal distance for males than for females (Roy et al., 2014). In western gorillas, conflicting genetic results were reported on male dispersal patterns. One study identified genetic networks among males (with males being more related to neighboring males than to distant ones) (Bradley et al., 2004), advocating for limited male dispersal. However, two other studies, both at similar and larger geographical scale (6,000 km^2^ compared to the previously mentioned study of 30 km^2^), found a single undifferentiated population based on Y‐chromosome microsatellite markers, thus consistent with extensive male dispersal (Douadi et al., 2007; Inoue et al., 2013).

For females, natal dispersal always occurs before the first reproductive event; secondary dispersal can occur soon after that or later in their reproductive life (Manguette, Robbins et al., 2020; Stokes et al., 2003). Previous studies suggested females immigrate preferentially into smaller breeding groups, selecting nascent units with younger and stronger silverbacks to avoid feeding competition and disease, and/or to increase protection and reduce attraction by infanticidal males or predators (Manguette, Robbins et al., 2020; Stokes et al., 2003). Transfers depend on various factors, including group size, group age, male phenotypic traits such as crest size (Baudouin et al., 2019; Breuer et al., 2012; Manguette, Robbins et al., 2020; Stokes et al., 2003) and loss of infants (Bai Hokou, long‐term data). In mountain gorillas, female relationships are clearly stronger among related females than among nonrelated females of the same group (Watts, 1994). If kin associations are also important in western gorillas, it could be hypothesized that female dispersal preserves these associations, either through single female dispersals toward groups that include related females or through co‐transfer of related females to the same group. Indeed, multifemale transfers between groups have been documented during both natal and secondary dispersals in western gorillas (Manguette et al., 2020; Stokes et al., 2003). At the same time, females are expected to avoid groups led by related males, in order to avoid consanguinity (Bradley et al., 2007).

Bradley et al. (2007) tested the hypothesis of female gorillas favoring groups with female kin in unhabituated groups and found that the average within‐group relatedness among females was higher than expected under a model of random dispersal. This result suggested that female kin associations occur during transfers or that females preferentially disperse to groups with female kin (Arandjelovic et al., 2014; Douadi et al., 2007). In parallel, the authors also found that the average relatedness of females to their group silverback was lower than expected, advocating the hypothesis that females avoid related males when dispersing. In mountain gorillas, it has been observed that female pairs are on average genetically more related within groups than among groups, yet male–female pairs were counter‐intuitively found to be genetically more related within groups than among groups (Roy et al., 2014). In western gorillas, the higher‐than‐expected level of female relatedness within groups inferred by Bradley et al.’s (2007) and Arandjelovic et al.’s (2014) study contrasts not only with other studies (Douadi et al., 2007; Inoue et al. 2013) but also with field observations that show very few social interactions among females or adults in general, either affiliative or even competitive (Doran‐Sheehy et al., 2009; Stokes, 2004; Masi 2020). Grooming and other physical affiliative behaviors have not been much recorded among wild western gorilla adults (Masi et al., 2009; Masi, 2020), raising the question of whether adult females have or not inclusive fitness benefits to having close kin in the same group.

Importantly, given the difficulties of habituating western gorillas (Doran‐Sheehy et al., 2007), all previous genetic studies in this species were based on fecal samples of unidentified individuals collected at nest sites (Arandjelovic et al., 2014; Bradley et al., 2007; Douadi et al., 2007). However, doing so results in potential individual misidentification, in particular in misidentifying predispersal subadult females as adult females. Moreover, all group members will not necessarily be sampled, which can result in higher or lower average relatedness by chance or sampling bias. These biases are particularly problematic when investigating within‐group female relatedness. Indeed, it is very difficult to know, from nest data alone, whether a female is an adult or a predispersal adolescent female, given that their body and dung size are roughly the same; and a predispersal adolescent female will inherently be strongly related to at least another group female, if her mother is still present. To get around this, some studies (Arandjelovic et al., 2014; Bradley et al., 2004) have combined multiple criteria, such as dung size, additional presence of infant feces in the nest, and absence of genetic relatedness with the silverback.

Here we analyzed, for the first time, genetic data from several groups of western gorillas (*G. gorilla*) in Central Africa. The study groups were habituated, providing the opportunity to identify with certainty the age/sex classes of dung samples, to sample nearly exhaustively all individuals, as well as to compare genetic data with behavioral observations and to make inferences about an individual's putative parents.

**FIGURE 1:**
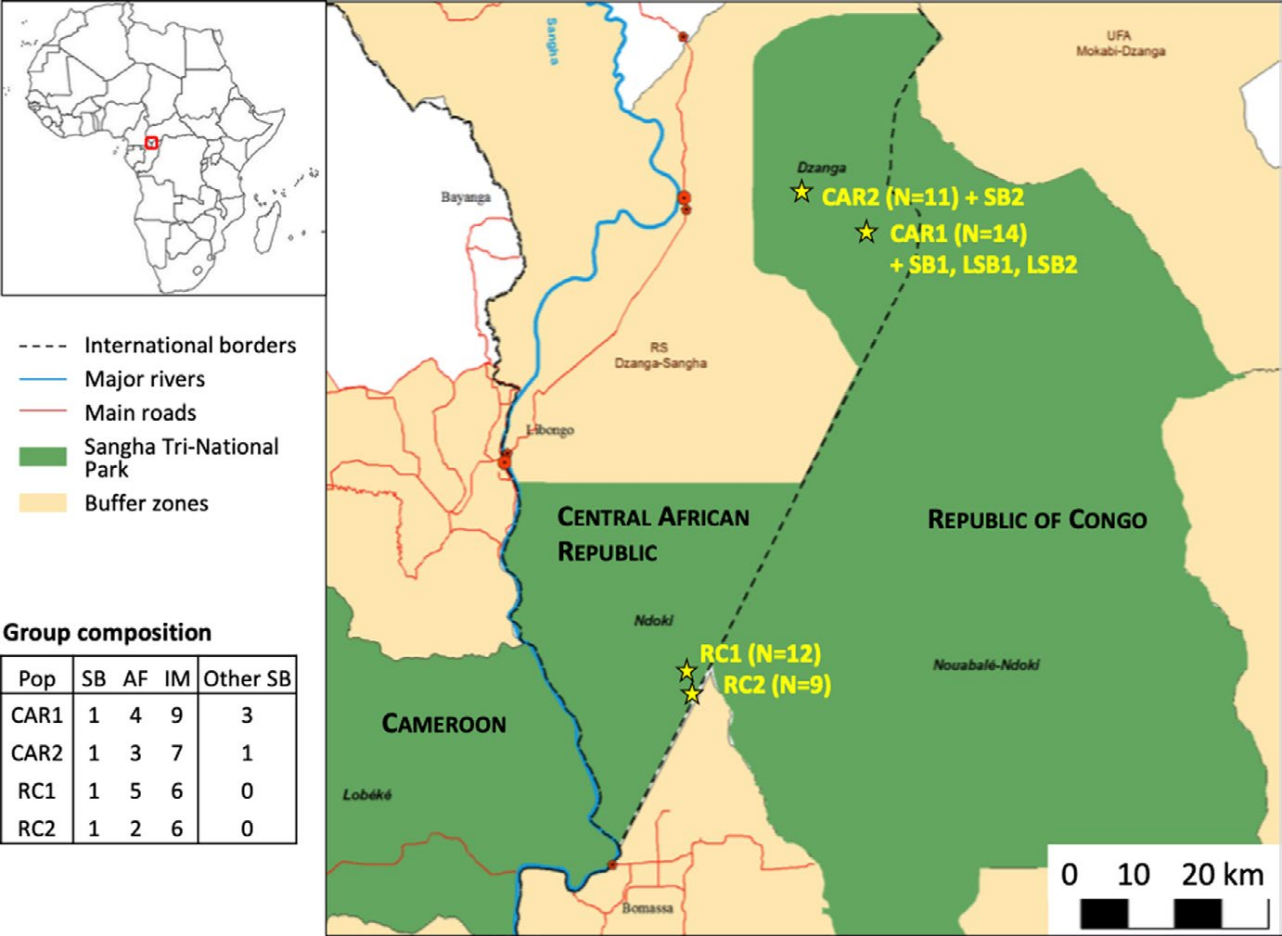
Spatial distribution of the study groups, with the group compositions of sampled individuals. CAR1/CAR2 are groups sampled at two field sites in Central African Republic (CAR), and RC1/RC2 are groups sampled at one field site in Republic of Congo (RC). The number of individuals sampled in each group is written in parenthesis (note that one individual from RC1 did not produce enough genetic data so was removed from the dataset for the genetic analyses). Pop, Population; SB, Silverbacks; AF, Adult Females; IM, Immatures, this includes all offspring from each study group, thus young silverbacks, blackbacks, subadults, juveniles and infants; Other SB, corresponds to two solitary silverbacks and two silverbacks from semi‐habituated groups

To investigate relatedness between males and females within and among groups ranging at different distances from each other, we collected fecal samples from 50 individuals in an area of approximately 110 km^2^ spread over a maximum distance of 70 km (Figure 1). Specifically, we investigated whether adult breeding females are (a) more related to within‐group females than those from other groups to favor cooperation (as suggested by Arandjelovic et al., 2014; Bradley et al., 2007) and (b) less related to their group silverback than to other silverbacks to limit inbreeding (as suggested by Bradley et al., 2007). We then investigated whether genetic relatedness influenced within‐group affiliative behavior among adult females (measured by spatial proximity), since kinship frequently coincides with proximity patterns and affiliative behavior (e.g., Kapsalis & Berman, 1996). Last, we compared genetic differentiation in males and females in relation to their geographic proximity. We expected males to disperse over greater distances, given that, unlike females, male gorillas spend at least part of their life history ranging as solitary individuals in search of (unrelated) females (Breuer et al., 2010; Parnell, 2002). This is expected to result in less genetic structure than for females within the same geographic area (Douadi et al., 2007; Roy et al., 2014). Investigating sex‐specific spatial genetic structure is crucial to increase our understanding of dispersal dynamics of the species.

## 2 METHODS

### 2.1 Study site, sample, and behavioral data collection

Our study was carried out at three field sites within the Sangha Trinational protected area complex (https://whc.unesco.org/en/list/1380): (a) Bai Hokou (N 2° 51.574', E 16° 28.045'; Datum: WGS84), (b) Mongambe (N 2° 55.077'; E 16° 23.324', Datum: WGS84) in Dzanga‐Ndoki National Park (DNNP, 1,222 km^2^) in the south‐western part of the Central African Republic (CAR), and (c) Mondika (N 4° 39.000'; E 18° 56.000', Datum: WGS84) in the Djeke Triangle west of Nouabalé‐Ndoki National Park in the Republic of Congo (RC) bordering DNNP. The distance between Bai Hokou and Mongambe in CAR and Mondika is approximately 57 and 61 km, respectively, across contiguous forest. The Sangha Trinational largely consists of semi‐deciduous rainforest (Harris, 2002) with a seasonal climate and a dry season (<100 mm monthly rainfall) between December and February and a peak rainy season between September and October.

We collected fecal samples from 50 individuals, of which 46 belonged to four habituated groups of western gorillas: 14 individuals from one group at Bai Hokou (named CAR1), 11 individuals from one group at Mongambe (CAR2), and 12 and 9 individuals, respectively, from two groups at Mondika (RC1 and RC2) (Figure 1). The two habituated groups in CAR were 9.1 km apart, and the two RC habituated groups had overlapping home ranges (Figure 1). We therefore sampled 46 individuals out of the 54 who composed the study groups throughout the study period. Samples from CAR1 group were collected between 2008 and 2017, those from CAR2 between 2011 and 2017, while samples from both RC groups were collected in 2014. During these study periods, we recorded the date and the identity of immigrant and emigrant individuals. The group composition changed over time mostly in terms of offspring or dispersing individuals, but little changes occurred in terms of acquisition of new adult females who then reproduced in the groups (one female and two females acquired in CAR‐1 and RC‐1 groups, respectively). The study group compositions are indicated in Figure 1 for the sampled individuals and in Table S1 for all individuals. We used age classes from Breuer et al. (2009).

To increase the adult male sample size (given western gorillas’ one‐male social system), we also sampled, between 2008 and 2017, the fecal samples of two silverbacks from two semi‐habituated groups and two unhabituated solitary silverbacks within the home range of two CAR habituated groups. The home range of the two silverbacks from the semi‐habituated groups (SB1 and SB2) partially overlapped with those of CAR1 and CAR2, respectively, while the two solitary silverbacks (LSB1 and LSB2) ranged within the home range of CAR1 and SB1 (Figure 1). Fecal samples were collected by Sh.M., T.F., and E.M. from identified individuals immediately after defecation during continuous focal follows (Altmann, 1974) and preserved in the field via the two‐step method (ethanol‐silica gel as described in (Arandjelovic et al., 2009).

Behavioral data collection included half‐day and full‐day observations of adult females between April to June 2008, June to August 2017, and June to July 2019 for the CAR groups, and April to May 2014 for the RC groups (female focal follows: *N* = 46 days and *N* = 11,536 total min; *N* = 520 scans with female–female distance). Behavioral data for this study were collected only in the frugivory season (see Masi et al., 2009, 2015 for seasonal definition) to control for variation in interindividual distances in relation to changes in fruit availability (Masi et al., 2009). Spatial proximity data among the adult females of each of the four habituated groups were collected by Sh.M., A.S.M., and S.M. for CAR groups, and A.S.G. and E.M. for RC groups (hours of focal sampling per each adult female dyad are provided in Table S2). The distance of the focal adult female to any other visible adult females of the group was collected using instantaneous scan sampling at 10‐min intervals (Altmann, 1974); for methods see (Masi & Breuer, 2018; Masi et al., 2009, 2012). For each scan, the distance was recorded according to three spatial categories: 0–5m, 6–10 m, and ≥10 m. If in a scan, the distance from the nonfocal female was not recorded, a distance of more than >10 m was assigned.

Western gorillas are listed as critically endangered by IUCN, and all samples were collected noninvasively under governmental authorization by Ministries of Education and Water and Forests of the CAR government and the Ministry of Scientific Research and Ministry of Forest Economy of RC. This research adhered to ethics and healthy protocols and legal requirements of the governments of both CAR and RC. All applicable international, national, and/or institutional guidelines for the care and use of animals were followed.

### 2.2 Geographic distance between the study groups

Geographical distances between the different study groups were calculated using the GPS coordinates of the center point of each habituated group's home range, which in turn were determined from long‐term data of each group's ranging patterns (2–4 years depending on the study group). Since the fecal samples of the two lone silverbacks and the two semi‐habituated silverbacks were collected within the home range of either the CAR1 or the CAR2 group (see Figure 1), we used the coordinates of the corresponding group for these silverbacks. CAR and RC groups belonged to a continuous population spread across those two countries, and no geographic barriers were present between them.

### 2.3 DNA extraction and genotype

Genomic DNA was extracted from 60 fecal samples, corresponding to 50 individuals (five individuals were extracted twice, using an additional fecal sample, due to poor quality data from the first extraction; and five fecal samples corresponded to already typed individuals). We used the QIAamp PowerFecal DNA kit (QIAGEN, USA), with approximately 100mg of fecal material. Based on previous literature (Arandjelovic et al., 2009, 2011; Bradley et al., 2000; Fünfstück et al., 2014; Roy et al., 2014), we chose 10 autosomal microsatellite loci (D2s1326, D4s1627, D5s1470, D6s1056, D7s817, D8s1106, D10s1432, D14s306, D16s2624, vWF), in addition to the amelogenin locus to identify the sex. We modified three primers in order to better match the gorilla genome (see Table S3). PCR amplifications were performed in a final volume of 20 µl composed of 0.5 U of Taq polymerase, 125 nM of each primer, 200 µM of dNTPs, 1× of buffer, and 1µl of extracted DNA. The reactions were performed in an Eppendorf Mastercycler with an initial denaturation step at 94°C for 5 min, followed by 36 cycles at 94°C for 30 s, 55°C for 30 s, 72°C for 20 s, and a final extension of 72°C for 10 min. Loci genotyping was realized with two sequencing multiplexes of six and five STRs, respectively (Table S3). Each genotype was obtained from at least three independent PCRs. Forward primers were fluorescently labeled, and reactions were further analyzed by capillary electrophoresis (ABI 310, Applied Biosystems). We used the software package GeneMarker (SoftGenetics LLC) to obtain allele sizes from the PCR product analysis. We chose to keep all individuals with more than six valid STRs, resulting in the removal of one individual (in the RC1 group). We thus had a final dataset of 49 individuals, corresponding to 22 adults and 27 infants/juveniles (i.e., one immature was removed).

### 2.4 Genetic inferences of parent–offspring relationships

We genetically assessed the parent–offspring relationships of every immature present in the samples using two methods, Cervus 3.0.7 (Kalinowski et al., 2007) and RELPAIR 2.0.1 (Epstein et al., 2000), in order to assess these relationships with more certainty. We also investigated this relationship for an adult female (RC1‐F4), who transferred from the RC2 group into the RC1 group while under observation. RELPAIR uses a maximum likelihood method according to the allele frequencies in the population that looks separately for maternities and paternities. We chose to run this software including all adults and each offspring, one by one, as well as including adults only, and we retained all relationships that came out as significant with a likelihood ratio higher than 10. For Cervus, we performed first a parentage analysis aiming at inferring jointly the father and mother of offspring, considering all adult females as potential mothers and all adult males as potential fathers. Separate paternity and maternity analyses were then performed for the offspring for which both parents could not be jointly identified. We assumed that 50% of candidate parents had been sampled and used a typing error rate of 1%, with 80% and 95% for relaxed and strict level of confidence, respectively. Confidence levels were computed based on the likelihood scores, using the standard simulation procedure developed in Cervus. We recorded also the number of mismatches between parents and offspring in each case.

### 2.5 Calculation of relatedness estimators and population differentiation among adults

We calculated two estimators of relatedness: QG (Queller & Goodnight, 1989) and LR (Lynch & Ritland, 1999), which are both method‐of‐moment estimators and perform better with data including five to twenty STRs (Csilléry et al., 2006). We used Kingroup (Konovalov et al., 2004) to calculate these estimators between all pairs of adult individuals and their associated p‐values (obtained by reshuffling sample alleles at each locus). In addition, we also estimated a kinship coefficient among all pairs of adult individuals using the Loiselle estimator (Loiselle et al., 1995), as implemented in SpaGeDi 1.5d (Hardy & Vekemans, 2002). Using the permutation analysis procedure implemented in the function *grouprel* of the *related* R package available at https://github.com/timothyfrasier/related, we determined whether females were significantly more related within each of the four groups than across groups. We then grouped all individuals by country (RC versus CAR) and assessed separately for males and females whether they were significantly more related within each country than among them. We determined also whether females were significantly more related to their silverback than to the other males in the population, using our own R script. We performed 10,000 permutations in each case.

We also estimated fixation indices among populations to determine the genetic structure of the different groups. We first grouped the two RC populations and the two CAR populations, respectively, and calculated a *R*_ST_ between countries, knowing that this index is specifically designed for microsatellite markers (Slatkin, 1995). We tested whether the *R*_ST_ significantly differed from zero by performing 10,000 permutations of individuals among all populations. Then, we computed all pairwise *R*_ST_ values between all four groups and regressed the pairwise values of *R*_ST_/(1‐*R*_ST_) against the logarithm of the distance. A positive regression slope is expected under a two‐dimensional isolation‐by‐distance (IBD) model (i.e., the greater the geographical distance, the higher the genetic distance; Rousset, 1997). The significance level of this slope was assessed by performing 10,000 permutations of population locations among all populations, which is equivalent to a Mantel test. All *R*_ST_ analyses were performed with SpaGeDi 1.5d, considering either all adult individuals or all adult females only. We could not perform the analyses on all adult male individuals, as there were only eight male individuals in total, with two groups containing only one male individual.

## 3 RESULTS

We successfully obtained genotypes at 10 microsatellites for 49 out of the 50 sampled western gorillas: 45 individuals belonging to four habituated groups, two silverbacks from semi‐habituated groups, and two lone silverbacks (Figure 1). All pairs of individuals had at least five markers in common. The 10 microsatellites had an average number of alleles of 6.8, with an average heterozygosity *H*_e_ of 0.751 (Table S3). The probability of identity (PID), that is, the probability for two individuals of having the same genotype, estimated with Cervus, was of 1.66 × 10^−10^.

### 3.1 Inferring parent–offspring relationships

Family relationships were inferred by long‐term observations on each of the study groups, since their habituation. We first inferred the parent–offspring relationships from behavioral data. The silverback of each group was always assumed to be the father of all offspring of that group. Mothers were inferred either because they were observed giving birth, lactating, or for elder offspring; maternity was based on the combination of different affiliative behaviors (e.g., spatial proximity, feeding, and social tolerance) and physical traits (e.g., nose print). The first step of our analysis was to compare these relationships inferred from behavioral data to those inferred from genetic data. Among the 28 individuals that we analyzed (27 immatures together with a mature female, RC1‐F4, who transferred from RC2 to RC1), we were able to genetically identify both parents for 20 offspring with Cervus (16 individuals at the 95% level and four individuals at the 80% level) and 15 offspring with RELPAIR (Table S4). Among the eight remaining cases, we separately assigned the mother for three individuals and the father for seven individuals with Cervus. Five of these seven individuals belonged to the RC2 group. Overall, among the 50 relationships where Cervus made a reliable inference (40 for RELPAIR; Table S4), only two discrepancies between the inferred relationships from field observations and the genetic data were identified and were similar for both Cervus and RELPAIR. These discrepancies corresponded to the paternity of two males in the groups CAR1‐IN6 and RC2‐IN2 (in both cases the eldest offspring of their group). In both cases, the solitary silverback LSB2 was identified to be more likely the father than the silverback of their respective groups. This was particularly significant for CAR1‐IN6 (significance level of 95% with Cervus, and a likelihood ratio ten times higher for the solitary silverback being the father compared to the group silverback with RELPAIR; Table S4), even if the silverback is also compatible with the offspring. Conversely, for RC2‐IN2, the paternity attribution to LSB2 was only significant at the 80% level. This inferred paternity is, therefore, more doubtful, the true father being possibly an unsampled male. In any case, this offspring was quite unlikely to have been sired by its group silverback, since they were not compatible at two loci. Finally, the adult female RC1‐F4 was found to be the daughter of the RC2 group silverback (by Cervus) and an adult female (RC2‐F1) of the same group (by Cervus and RELPAIR), as predicted from field observations of the natal transfer.

### 3.2 Contrasting intra‐ versus intergroups relatedness among adults

We first used RELPAIR to test for significant genetic relationships between adults. We found a significant full sibling relationship between two females from the CAR1 group (likelihood ratio of 41.5) and a parent–offspring relationship between two females from the two neighboring groups in RC (likelihood ratio of 29.1).

We then measured the QG and LR relatedness estimators, as well as the Loiselle kinship estimator and found a strong correlation between the three metrics (Table S5 and Figure S1, Pearson's coefficient: *r* = 0.841–0.948, *p* < .0001 for all three comparisons; *N* = 231 adult pairs). Given their similarity, and the fact that previous studies have shown that the QG estimator had a smaller variance for higher‐order relationships (Blouin, 2003; Csilléry et al., 2006), we decided to keep only the QG estimator for further analyses.

**FIGURE 2:**
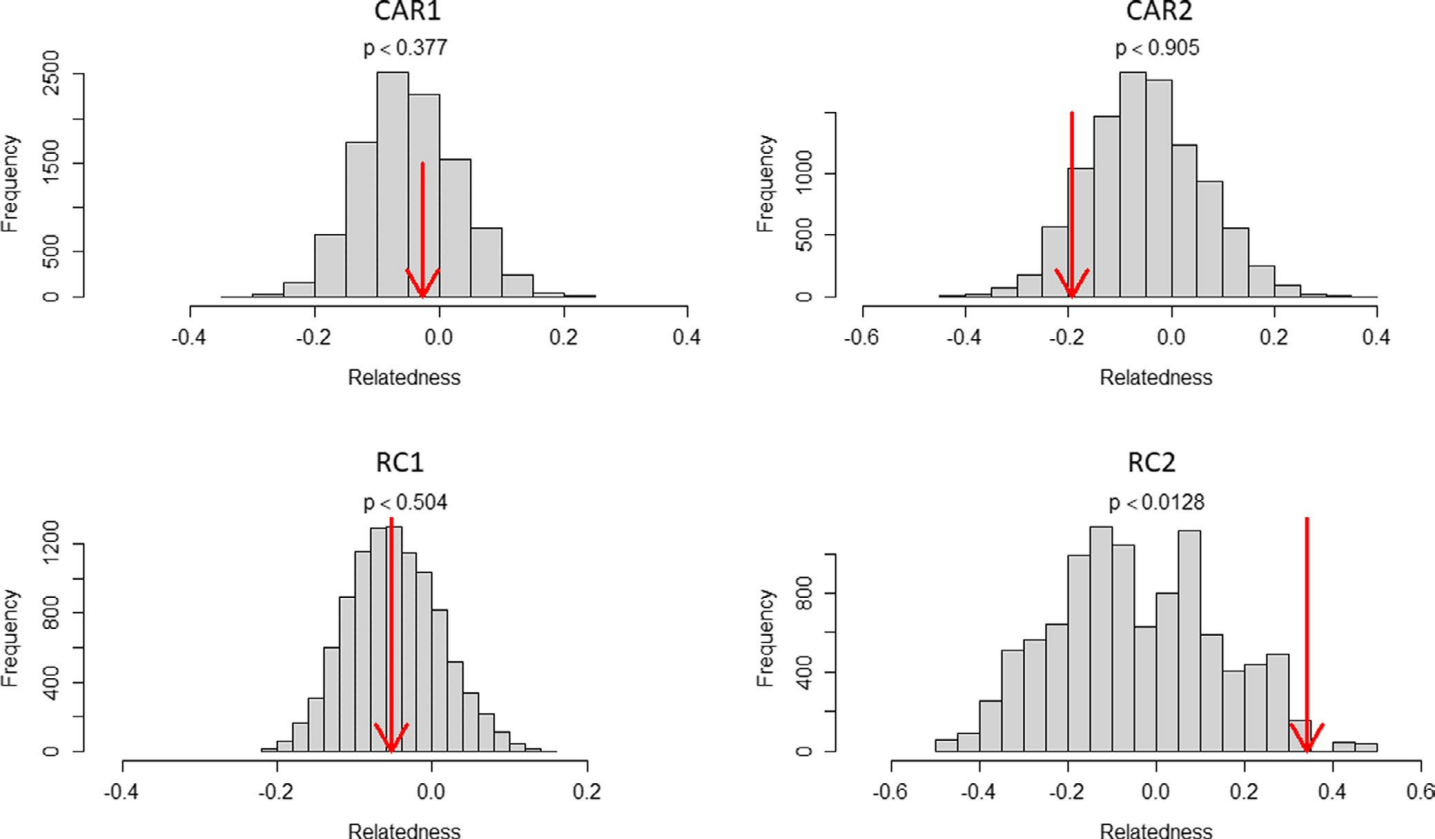
Histogram of the expected average relatedness values within each group obtained by 10,000 random permutations of individuals among groups. The red arrow indicates where the observed value lies. The *p*‐value was computed as the percentage of permutations where the expected values were greater than or equal to the observed value. All computations were performed with the function *grouprel* of the R package *related* (see *Methods*), and the graphs were also displayed using this function

We then investigated whether pairs of females within groups were more related than pairs of females taken at random in the whole population (Figure 2). We found that for three groups, there was not a significant excess of relatedness: CAR1 (*p* = .377), CAR2 (*p* = .905), and RC1 (*p* = .504), while it was significant for RC2 (*p* = .0128). Accordingly, we found that a similar proportion of female pairs was significantly related within groups (2/20 = 10%) and among groups (7/71 = 9.9%; Table S5). Among the seven pairs significantly related among groups, four corresponded to females from neighboring groups (three from RC and one from CAR, i.e., 2–10 km apart) and three to females from distant groups (all being between CAR1 or CAR2 and RC1, i.e., at 57–61 km from one another).

Moreover, we did not find that females were more related to the silverbacks of their group than to other silverbacks (*p* = .169), the average QG relatedness value between females and the silverback of their group being of −0.0112. Similar proportions of male‐female pairs were found to be significantly related within and among groups (1/14 = 7.1% and 9/98 = 9.2%, respectively; Table S5). Among the nine pairs significantly related but from different groups, seven belonged to distant groups (57–61 km apart); all seven of these pairs included one individual from CAR1 or CAR2 and one from RC1.

### 3.3 Influence of geographical distance on genetic differences

**FIGURE 3:**
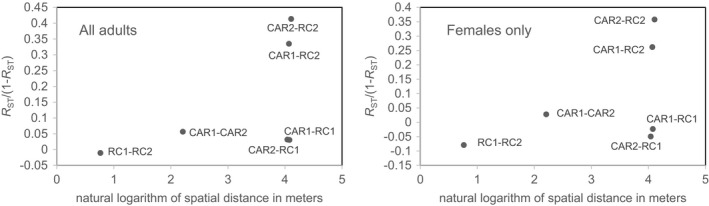
Pairwise values of the population differentiation index *R*_ST_/(1‐*R*_ST_) plotted against the logarithm of the geographical distance using all adults (left) or only female adults (right)

With regard to the population differentiation indices, we found that the *R*_ST_ value (better suited for microsatellite markers) was significant among distant groups (i.e., between RC and CAR groups, including all adults, *R*_ST_ = 0.0821, *p* = .0089). *R*_ST_ was however not significant when only females were included in the analysis, likely because of power issues due to a reduced sample size. Interestingly, we found a significant correlation between the *R*_ST_/(1‐*R*_ST_) coefficient and the logarithm of geographic distance, both when we considered all adults (regression slope *b* = 0.0682, *p* = .0399) or only the females (regression slope *b* = 0.0652, *p* = .042; Figure 3). However, we could not investigate this relationship in males, given the limited adult male sample size.

Further, when merging the two CAR groups and the two RC groups, respectively, we found that that RC females were more related than females taken at random in the whole population (*p* = .0201), while it was not the case for CAR females (*p* = .686). Conversely, pairs of males were not found to be more related within each country than pairs of males from different countries (*p* = .347 for CAR and *p* = .886 for RC). When looking in more details at the relatedness among the eight silverbacks, we found that two out of the 28 pairs were significantly related: one between neighboring silverbacks (CAR2 and LSB2, at 10 km apart) and one between distant silverbacks (CAR1 and RC1, 57 km apart; Table S5).

### 3.4 Adult female spatial and genetic proximity

For 21 pairs of females belonging to the same group, we obtained both genetic relatedness estimators and spatial proximity data from field observations. In 38% of adult female dyad scans (*N* = 520), adult females were greater than 10 m from each other, in 33% of scans between 6 and 10 m, and in 29% of scans between 0.5 and 5 m. They were never found at less than 0.5 m away from each other or in physical contact. Overall, the spatial proximity between dyads of adult females (average number of dyad scans <10 m corrected for the number of hours of focal dyad sampling) did not correlate with their QG genetic relatedness (Spearman correlation *r* = 0.144, *p* = .594, *N* = 21; Figure S2).

## 4 DISCUSSION

### 4.1 Insights on western gorilla socioecology from paternity analysis

The two methods used to genetically reconstruct the familial relationships (Cervus and RELPAIR) were largely consistent with each other, with more power using Cervus compared to RELPAIR. In the majority of cases (25/27 using Cervus and 20/22 using RELPAIR), paternity analysis identified the group silverback as the father of the group's immature offspring. Nevertheless, both methods showed that in two of the study groups (CAR1 and RC2), the eldest nonadult was likely not the son of the group silverback, as also found in two previous studies on unhabituated groups (Arandjelovic et al., 2014; Hagemann et al., 2018).

Further investigations are needed to better understand why exceptions to the exclusive paternity of the silverback occur in this one‐male polygynous species and under which social contexts. This is possible via transfer of pregnant females (Manguette, Breuer et al., 2020), with nonadults joining groups after group disintegrations, or through extragroup paternity, in which case it might suggest that even if the silverback of the group is siring the majority of the offspring, other males could contribute occasionally to reproduction, similarly to that observed in multimale groups of mountain gorillas (Bradley et al., 2005). However, from the literature, group formation was largely made by solitary males acquiring a harem (Breuer et al., 2010) and no evidence exists for group takeovers from other silverbacks. Importantly, if extragroup paternity does occasionally occur in western gorillas, the absence of a paternal relationship between the group silverback and a given group female cannot be reliably used to identify adult females or exclude them as offspring, as done in previous studies on unhabituated groups of this species (Arandjelovic et al., 2014; Bradley et al., 2007; Douadi et al., 2007). We observed here extragroup paternity only for male offspring, but it cannot be excluded that it may occur for female offspring in other instances.

The genetic analysis also provided insights on group age. Most immatures for which Cervus could reliably identify the father but not the mother were in the same group, RC2, which suggests that their mothers may have already left the group, and females have been found to leave older or weaker males to join younger (fitter) males (Baudouin et al., 2019; Manguette, Robbins et al., 2020). Indeed, the RC2 group composition (with very few adult females and quite a few older offspring) was in agreement with that of previously described old groups (Parnell, 2002). This is also consistent with the silverback´s physical appearance (e.g., deflated crest, Breuer, personal observation). Thus, using genetic analysis during the habituation of a new gorilla group might not only help in assessing if the same group is being followed over time (Bradley et al., 2008), but also in determining whether the target group is of a suitable age to undergo habituation, by assessing whether the mothers of the immatures are still present in the group.

The adult female RC1‐F4 was found to be the daughter of the silverback and an adult female of the RC2 group, as predicted from field observations (i.e., she was observed transferring from RC1 to RC2). This result provides an additional case to the body of evidence that natal transfers are more likely to occur between neighboring groups. This could eventually lead to an excess of within‐group female relatedness (as observed in Bradley et al., 2007), given that dispersal is not random but proportional to the geographic distance, and thus, the genetic distance in the case of females. Female dispersal allows avoiding inbreeding, which could lead to the apparition of deleterious traits. Moreover, it improves reproductive success by limiting intragroup feeding competition. Therefore, western gorilla females seem to show unconditionally a strategy of natal dispersal by departing from the group in which they were born (Baudouin et al., 2019; Manguette, Robbins et al., 2020; Stokes et al., 2003).

### 4.2 Intra‐ versus intergroup relatedness among females: insights into dispersal strategy

Among all postdispersing females, we found only two pairs of female siblings among the four habituated groups. Even though we found one pair of full sisters within the CAR1 group, the other female pair with a high level of relatedness was found among neighboring groups in RC. Apart from these two cases, permutation analysis showed that adult females were generally not more related within groups than expected at random (Figure 2), except for the RC2 group, which consisted only of two related females. While we cannot exclude that this lack of significant excess in within‐group relatedness may be linked to some extent with the limits of our datasets (49 individuals, 10 microsatellites), our result is nevertheless consistent with field observations. Indeed, while co‐transfers of adult females have been observed (Manguette, Breuer et al., 2020), they are rare, as in western gorillas, the timing of female voluntary transfer depends on the age of the offspring and male coercive strategies (Breuer et al., 2016; Harcourt & Stewart, 2007b; Manguette et al., 2019; Stokes et al., 2003). Our result is however in contrast with some previous results found in western gorillas (Arandjelovic et al., 2014; Bradley et al., 2007), but in line with others (Douadi et al., 2007; Inoue et al. 2013). These contrasting results may have resulted from biases in sampling nonhabituated gorillas from nest sites, that is, errors in attributing females to immature versus mature classes based on analysis of their genetic relationship with the silverback, their dung size, and the presence of infants fecal samples in their nest (Bradley et al., 2008). Indeed, if a mature female has an immature daughter that is wrongfully classified as mature, this will lead to an artificial increase of the average kinship level among mature females in the group.

Our general lack of kin association among adult female western gorillas within the same group is more in keeping with the hypothesis that nonphilopatric females are not expected to cooperate (Harcourt & Stewart, 2007b; Robbins & Robbins, 2018; Sterck et al., 1997; Watts, 1994). Field observations also corroborate this, showing little or no affiliative behavior among adult females within the same western gorilla group (Stokes, 2004; Masi 2020). Our results show that adult females spend little time in close proximity to each other (29% of observations between 0.5–5 m and none <0.5 m) and that their spatial distance does not correlate with their genetic distance, unlike what was found in other species (Kapsalis & Berman, 1996).

Further, we found no evidence that female western gorillas avoid transferring to related silverbacks, as females did not appear to be more related to the silverbacks of their group than to other silverbacks, contrarily to findings on nonhabituated gorillas (Douadi et al., 2007) or on mountain gorillas (Vigilant et al., 2015).

### 4.3 Comparing genetic and geographical distances

**FIGURE 4:**
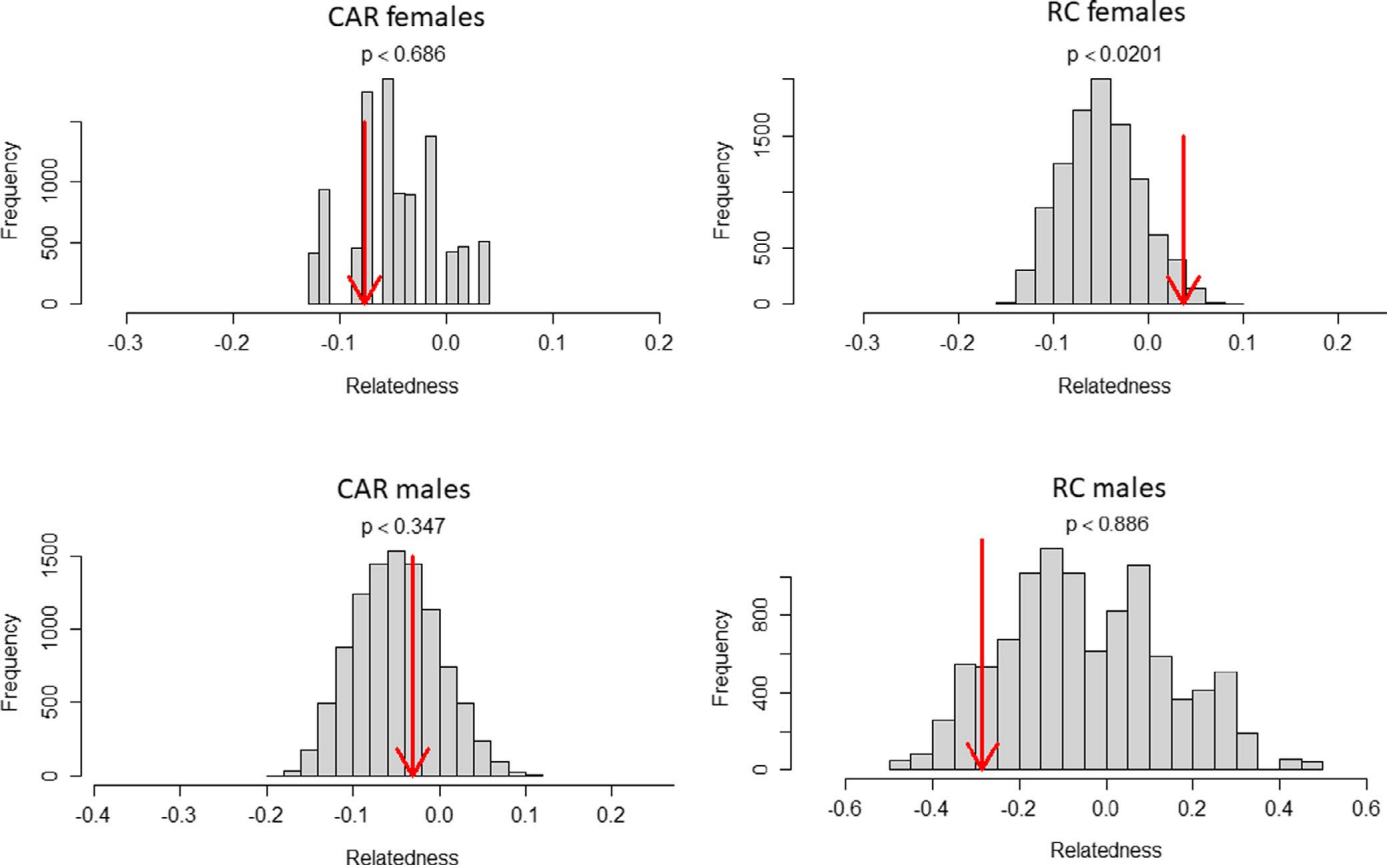
Histogram of the expected average relatedness values within each country obtained by 10,000 random permutations of individuals among countries, for females (upper) and for males (lower). The red arrow indicates where the observed value lies. The *p*‐value was computed as the percentage of permutations where the expected values were greater than or equal to the observed value. All computations were performed with the function *grouprel* of the R package *related* (see methods), and the graphs were also displayed using this function

Among the seven significantly related female pairs belonging to different groups, three pairs corresponded to females from the neighboring RC groups, with individuals apparently transferring between the two groups. In particular, as pointed out above, we showed that a mature female from the RC1 group was born in the RC2 group. However, three of the remaining related female pairs were from distant areas (between CAR and RC, 57–61 km apart), suggesting that multiple transfers of one or more related females dispersing further afield also occur occasionally, since females do not range alone (Breuer et al., 2010; Parnell, 2002; Stokes et al., 2003). The infrequency of such long‐distance female transfers is corroborated by the fact that relatedness was found to be significantly higher among females within RC than across countries (Figure 4). In contrast, we did not find such a result for males, indicating that males disperse further than females, as has been previously suggested (Douadi et al., 2007).

Our findings are also consistent with field observations on western gorillas. Females transfer from one group to a neighboring group during intergroup encounters which likely does not range far from their natal group (Manguette, Robbins et al., 2020; Stokes et al., 2003). Later, both voluntary (e.g., predispersal, in aging groups) and involuntary (e.g., as a consequence of silverback death or group disaggregation) secondary transfers allow them to disperse wider, increasing the likelihood that they will reside in a group with no or little kin (Manguette, Robbins et al., 2020; Stokes et al., 2003). In contrast, males often spend months to years as solitaries while they reach maturity and gain sufficient experience to attract and protect females to form their own breeding group (Breuer et al., 2009; Breuer et al. *in preparation*); some males may never succeed in acquiring females. Thus, males can disperse much further from their natal group than females, particularly during the male propedeutical solitary phase. This may also be true for those that never succeed in acquiring females or those that later lost their females. While this longer dispersal distance for males may not be consistent with the hypothesis of a “dispersed male network” (Bradley et al., 2004), this result is in line with a similar‐ and large‐scale study based on Y‐chromosomal microsatellite markers of unhabituated western gorillas that found a single undifferentiated male population (Douadi et al., 2007; Inoue et al., 2013).

In addition, using the population differentiation index *R*_ST_, we found that CAR and RC gorillas were genetically distinct, and we found a signal of isolation by distance as indicated by the significant relation observed between pairwise *R*_ST_ and geographical distance, whether considering all adults or females only (Figure 3). This is consistent with the clinical pattern found in a larger scale study also using microsatellite genotypes (~37,000 km^2^, Fünfstück et al., 2014). Our finding suggests thus again that, although males can disperse further than females, dispersal overall is limited in this species.

### 4.4 Comparison with other species—where do the western gorillas fit?

Our study showed that *Gorilla* is a genus where extragroup paternity is limited compared to other polygynous species such as lions (Lyke et al., 2013), which indicates a rather strict control of male gorillas over their group females, for example, via coercive behavior (Breuer et al., 2016). Moreover, as shown for mountain gorillas (Roy et al., 2014), we found a positive relation between geographical distances and genetic differentiation (Figure 3). While we did not find a higher‐than‐expected level of relatedness among females within groups, we found some evidence that dispersal by females is limited in this species (Figure 4) and that long‐distance dispersal is more likely in males, as in other polygynous species such as elephant seals (Fabiani et al., 2003) or lions (Curry et al., 2020; van Hooft et al., 2018). In fact, polygyny may favor male‐biased dispersal, as competition for females will lead to greater male dispersal, such as is seen in shore birds (D’Urban Jackson et al., 2017). Similarly, in other social animals, dispersing males occasionally transfer their genes over longer distances (Fabiani et al., 2003; Mech & Boitani, 2003).

## 5 CONCLUSION

Overall, comparing our findings with previous studies, all carried out on unhabituated groups, our study exemplifies how results can be affected by geographical scale and the incorrect categorization of individual samples. In our study on habituated groups, the vast majority of genetically inferred parent–offspring relationships matched field observations. Likewise, inferred paternities and maternities from genetic data were largely consistent with those inferred from field observations of gorillas residing in habituated groups. Together, our results strongly suggest that relatedness levels within and between sexes do not seem to be factors influencing female dispersal patterns in this species, contrarily to what was previously suggested (Bradley et al., 2007). Male reproductive strategies are rather the driver of dispersal in western gorillas—age and male fitness is key and impacts both male and female reproductive strategies, confirming previous studies (Breuer et al., 2012; Caillaud et al., 2008)—females pursue a strategy of secondary transfer and only rarely are able to confuse paternity (this study and Manguette, Breuer et al., 2020). Some exceptions and counterstrategies do exist but appear to be rare.

## CONFLICT OF INTEREST

The authors declare to have no conflicts of interest.

## AUTHOR CONTRIBUTION

**Shelly Masi:** Conceptualization (lead); Data curation (lead); Formal analysis (supporting); Funding acquisition (lead); Investigation (lead); Methodology (equal); Project administration (lead); Resources (lead); Supervision (equal); Validation (equal); Visualization (supporting); Writing‐original draft (lead); Writing‐review & editing (lead). **Frederic Austerlitz:** Conceptualization (supporting); Data curation (supporting); Formal analysis (lead); Investigation (equal); Methodology (equal); Software (lead); Validation (equal); Writing‐review & editing (equal). **Chloé Chabaud:** Formal analysis (supporting); Investigation (supporting); Methodology (supporting); Software (supporting); Visualization (supporting); Writing‐original draft (equal); Writing‐review & editing (supporting). **Sophie Lafosse:** Data curation (equal); Formal analysis (supporting); Funding acquisition (supporting); Resources (supporting). **Nina Marchi:** Data curation (equal); Formal analysis (supporting); Resources (supporting); Writing‐review & editing (supporting). **Myriam Georges:** Data curation (equal); Formal analysis (supporting); Resources (equal). **Françoise Dessarps:** Data curation (equal); Formal analysis (supporting); Resources (equal). **Silvia Miglietta:** Data curation (equal); Formal analysis (supporting); Resources (equal). **Andrea Sotto‐Mayor:** Data curation (equal); Formal analysis (supporting); Resources (equal). **Aurore San Galli:** Data curation (equal); Resources (equal). **Ellen Meulman:** Data curation (equal); Resources (equal). **Emmanuelle Pouydebat:** Data curation (equal); Funding acquisition (lead); Resources (supporting); Writing‐review & editing (supporting). **Sabrina Krief:** Data curation (equal); Funding acquisition (lead); Methodology (supporting); Resources (supporting); Writing‐review & editing (supporting). **Angelique Todd:** Data curation (supporting); Methodology (supporting); Resources (supporting); Writing‐review & editing (equal). **Terence Fuh:** Data curation (supporting); Methodology (supporting); Resources (supporting); Writing‐review & editing (supporting). **Thomas Breuer:** Data curation (supporting); Investigation (supporting); Methodology (supporting); Resources (supporting); Visualization (supporting); Writing‐review & editing (equal). **Laure Segurel:** Conceptualization (lead); Data curation (equal); Formal analysis (lead); Funding acquisition (equal); Investigation (lead); Methodology (lead); Project administration (equal); Resources (equal); Software (lead); Supervision (equal); Validation (lead); Visualization (lead); Writing‐original draft (equal); Writing‐review & editing (lead).

## Supporting information

Appendix S1

Fig S1

Fig S2

Table S1‐S5

## Data Availability

All genetic data are deposited in Dryad https://doi.org/10.5061/dryad.t4b8gtj1s. All genetic analyses were performed with publicly available programs.
