# Supplementary material for: No evidence for female kin association, indications for extragroup paternity, and sex‐biased dispersal patterns in wild western gorillas
Source: Ecol Evol. 2021 May 25;11(12):7634–46. doi: 10.1002/ece3.7596 (PMC8216920; doi:10.1002/ece3.7596)
Supplement: Supplementary file 1 — Appendix S1 [file ECE3-11-7634-s002.docx]

**Supplemental Information for:**

**No evidence for female kin association, indications for extra-group paternity and sex-biased dispersal patterns in wild western gorillas**

Short running title: **Genetic structure in wild western gorillas**

Shelly Masi1*, Frédéric Austerlitz1*, Chloé Chabaud1,2, Sophie Lafosse1, Nina Marchi1, Myriam Georges1, Françoise Dessarps-Freichey1, Silvia Miglietta1, Andrea Sotto-Mayor1, Aurore San Galli1, Ellen Meulman1, Emmanuelle Pouydebat3, Sabrina Krief1, Angelique Todd4, Terence Fuh4, Thomas Breuer5, Laure Ségurel1

1 Eco-anthropologie, Muséum national d’Histoire naturelle, CNRS, Université de Paris, Paris, France

2 Department of Biology, Ecole normale supérieure, PSL University Paris, F-­75005, Paris, France 3 UMR7179 MECADEV CNRS/MNHN, Department Adaptations du Vivant, 55 rue Buffon, Paris, France

4 Dzanga-Sangha Protected Areas, World Wide Fund for Nature, BP 1053 Bangui, Central African Republic

5 Wildlife Conservation Society, Global Conservation Program, 2300 Southern Boulevard, Bronx, NY 10460, USA Orcid: 0000-0002-8387-5712

*S. Masi and F. Austerlitz should be considered joint first author

Corresponding author: Shelly Masi [masi@mnhn.fr](mailto:masi@mnhn.fr)


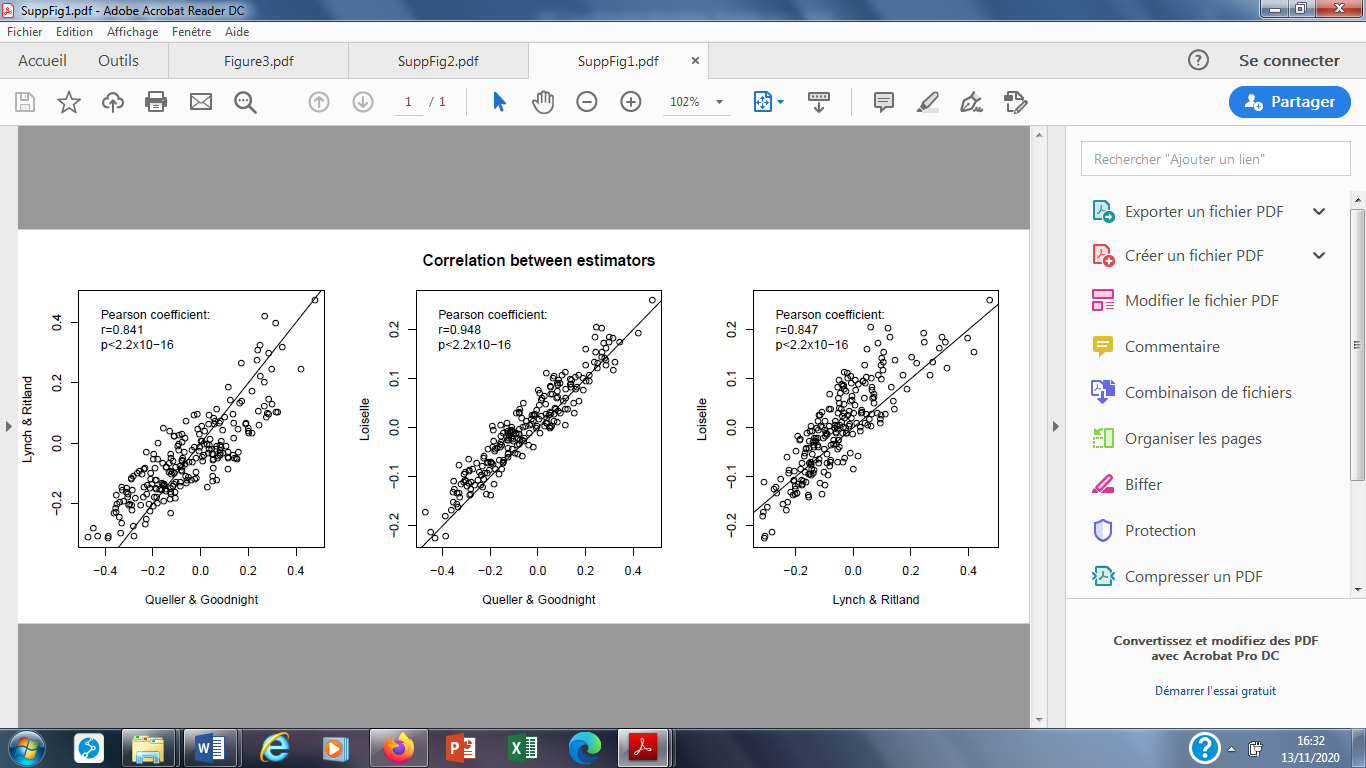


**Supp Fig 1.** Correlation between the three relatedness/kinship estimators (N = 231 pairs), as well as the Pearson coefficient and p-value. For the first graph, the line x = y is plotted, while for the last ones, the line x = 2y is plotted.


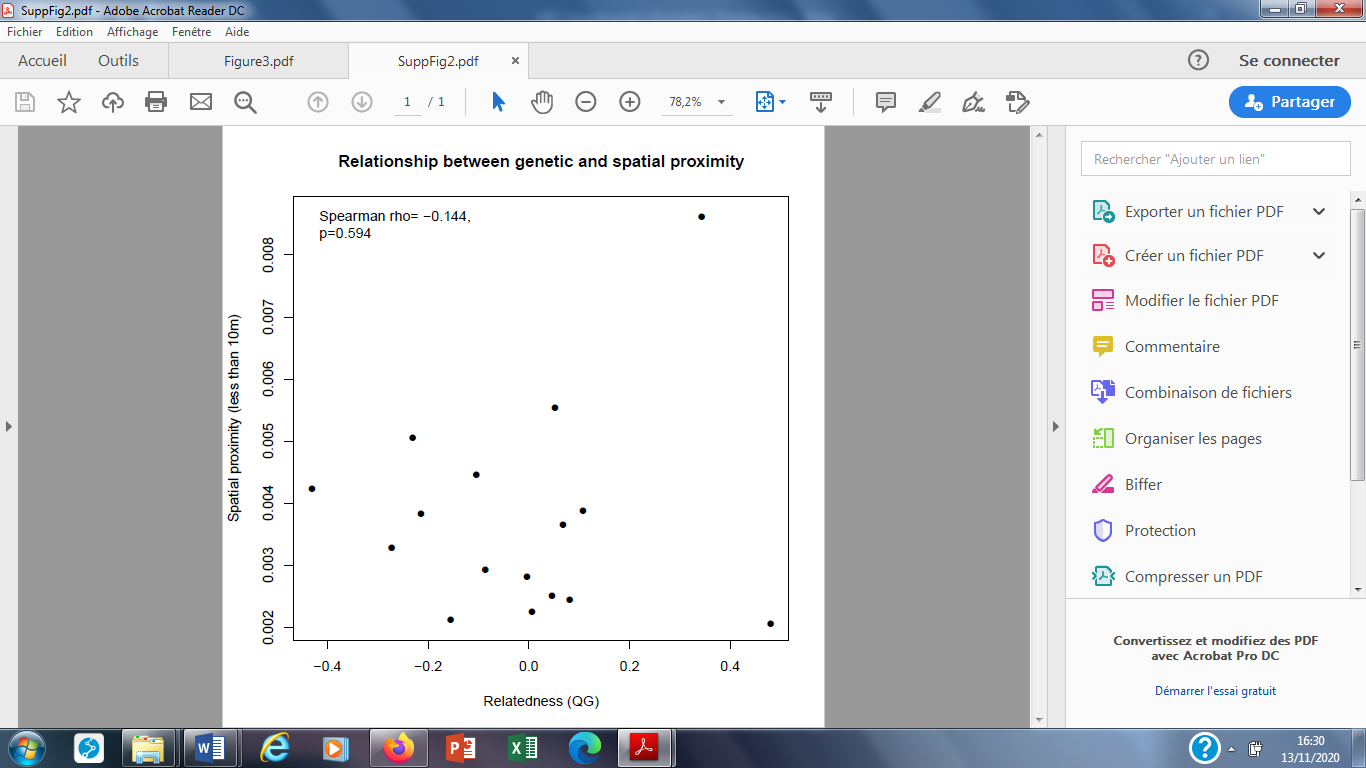


**Supp Fig 2.** Correlation between the QG relatedness estimator and the mean spatial proximity (for distances lower than 10 meters) for pairs of within-group adult females (N = 21).

**Table legends**

Tables are attached as excel files

**Supp Table 1.** Group compositions of the study groups and over time information about the study individuals.

**Supp Table 2.** Minutes of focal animal sampling per dyad of adult females within the same study group.

**Supp Table 3.** List of primers used, number of alleles and expected heterozygosity for each microsatellite marker. The base pairs modified to better match the gorilla genome sequence (gorGor5, Mar 2016) are underlined and bold.

Supp Table 4. Most likely candidate mothers and fathers inferred with Cervus and RELPAIR, along with the field observed mothers and fathers. For Cervus, “Trio confidence” indicates the confidence level of the parentage analysis, looking jointly for the mother and the father of the offspring. “Mother confidence” corresponds to the maternity analysis and “father confidence” to the paternity analysis. These analyses were performed only when a parental pair was not identified by the parentage analysis. * corresponds to 95% significance level, + to 80% significance level. The number of mismatches (incompatible loci between parent and offspring, probably due to genotyping errors) are indicated in each case. For RELPAIR, the likelihood ratio (LR) of a given relationship (PO=Parent/Offspring; FS=Full Siblings; AV=Avuncular relationship) as compared to "Unrelated" is written in parenthesis. Differences between the field observations and the genetic approaches are indicated in red. “?” indicate unsure identification from the field, either because the individual was not observed when being infant or juvenile or because the parent relationship is unknown.

**Supp Table 5**. Relatedness (Queller & Goodnight and Lynch & Ritland) and kinship (Loiselle) estimators between all pairs of adult individuals. FF=Female-Female; MF=Male-Female; MM=Male-Male; Inter=between groups; Intra=within groups
