# Supplementary figures and images for: No evidence for female kin association, indications for extragroup paternity, and sex‐biased dispersal patterns in wild western gorillas
Source: Ecol Evol. 2021 May 25;11(12):7634–46. doi: 10.1002/ece3.7596 (PMC8216920; doi:10.1002/ece3.7596)

## Correlation between estimators

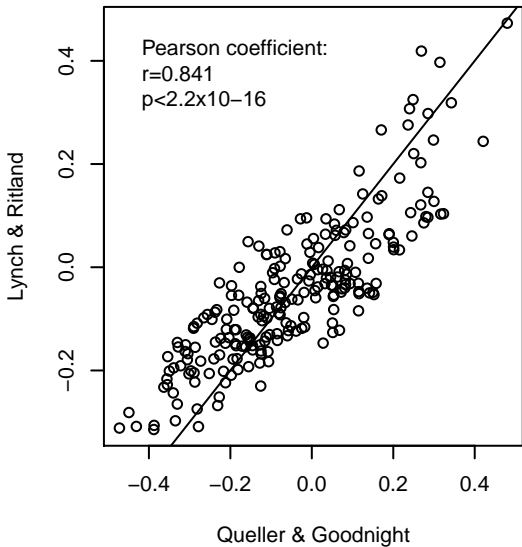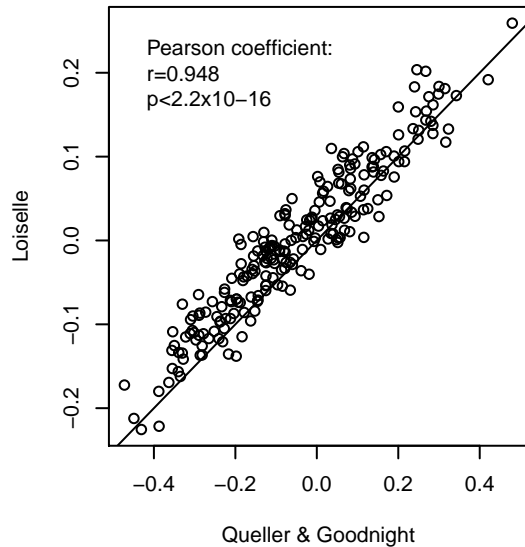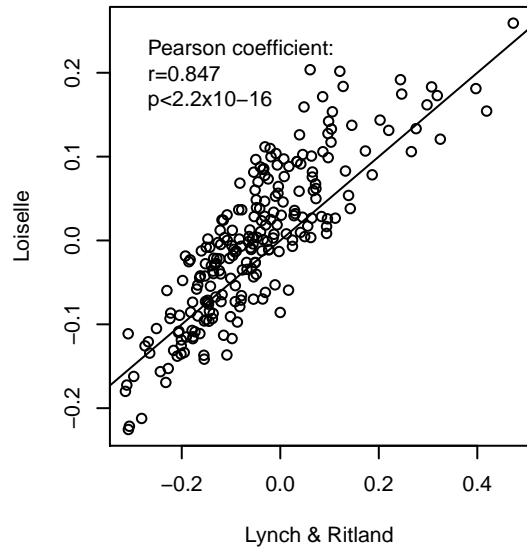

Supplement: Supplementary file 2 — Fig S1 [file ECE3-11-7634-s004.pdf]

## Relationship between genetic and spatial proximity

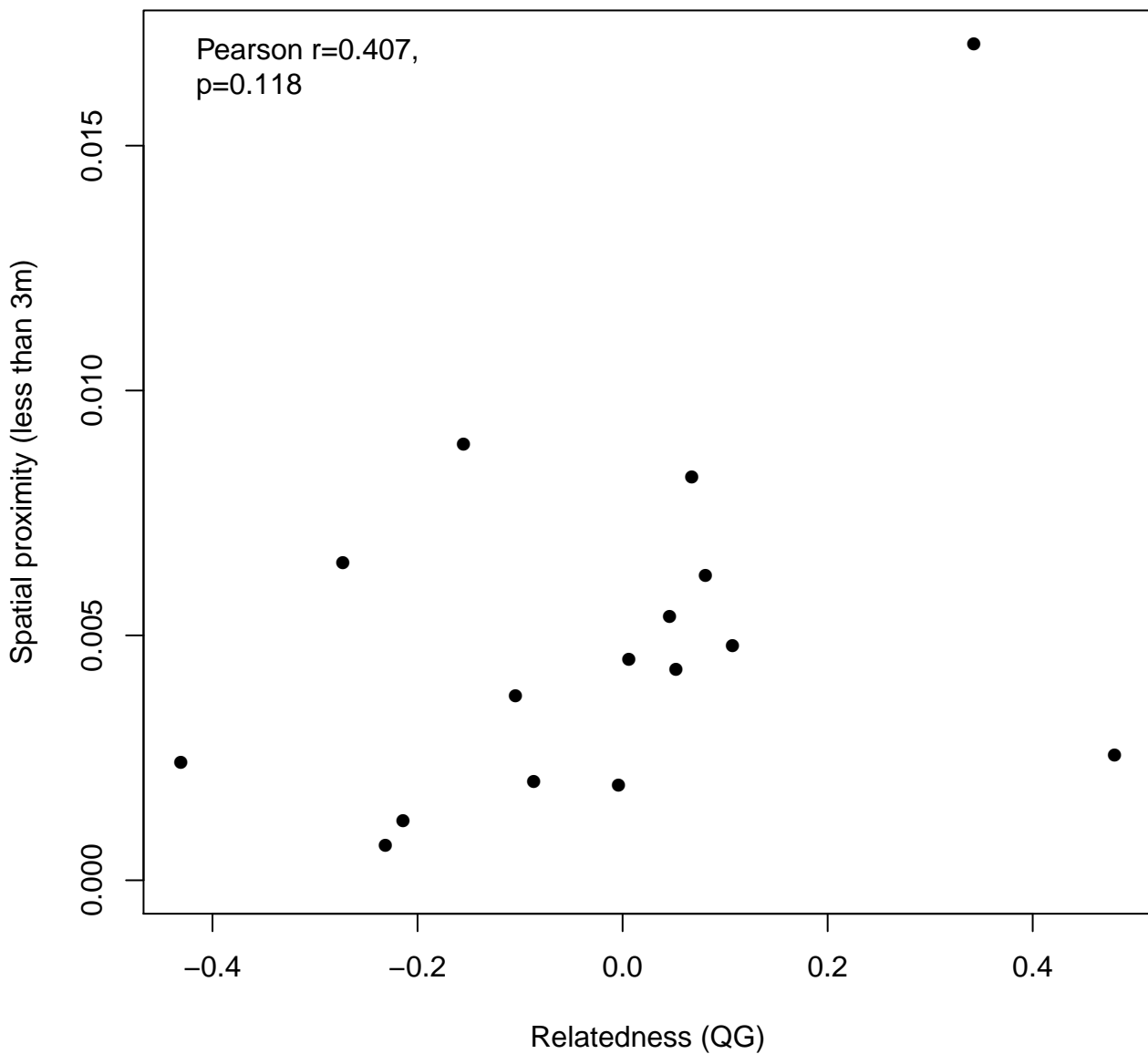

Supplement: Supplementary file 3 — Fig S2 [file ECE3-11-7634-s001.pdf]
